# Development of forensic blood substitute: Focusing on luminol reaction functionality

**DOI:** 10.1111/1556-4029.70018

**Published:** 2025-03-13

**Authors:** Sang‐Yoon Lee, Yeon‐Soo Jo, Hwa‐Seon Lim, Ki‐Jong Rhee

**Affiliations:** ^1^ Department of Forensic Sciences Yonsei University Wonju Republic of Korea; ^2^ Department of Forensic Sciences Sungkyunkwan University Suwon Republic of Korea

**Keywords:** artificial blood, bloodstain pattern, chemiluminescence, fluorescence, forensic blood substitute, luminol, luminol reaction, synthetic blood

## Abstract

Bloodstain pattern analysis (BPA) is essential for reconstructing crime scenes using the shape and distribution of bloodstains. Luminol, which emits blue light in a chemiluminescent reaction with hemoglobin, is commonly used to detect latent blood. Artificial blood substitutes, designed for luminol reactivity or bloodstain pattern reproduction, are widely used in forensic experiments. These artificial blood substitutes, including synthetic and spatter blood, are commercialized with distinct functionalities: luminol reactivity and bloodstain pattern reproduction, respectively. This study introduces a new blood substitute that combines both functionalities. In this study, we verified the luminol reaction functionality of a newly developed forensic blood substitute, which also demonstrates bloodstain pattern reproduction capabilities. Additionally, we assessed its morphological features by analyzing the coordinate characteristics of the area of origin in impact spatter patterns. The visual inspection, without the use of supplementary equipment, revealed that there was no noticeable difference in the luminol fluorescence reaction between the developed blood substitute and the four types of commercial synthetic blood. Furthermore, the developed blood substitute exhibited the smallest standard deviation, confirming it to be the most homogeneous and stable artificial blood substitute. Morphological analysis revealed that the new substitute closely mimics human blood, particularly in *z*‐axis coordinates. These findings establish the substitute as a versatile tool for forensic analysis, enhancing both luminol detection and bloodstain pattern reproduction.

**Highlights**

- YFBS mimics human blood, with remarkable similarities in luminol reactivity and spatter patterns.
- YFBS replicates the luminol reaction while preserving key rheological properties of real blood.
- YFBS shows minimal visual differences from other substitutes, enabling realistic applications.
- YFBS is ideal for studies needing both chemical and physical properties of real human blood.

## 1 INTRODUCTION

Crime scenes often contain various types of biological evidence, such as saliva, semen, and hair. Among these, blood is particularly crucial as it frequently indicates trauma and physical assault [1, 2], providing valuable insights into the sequence of events. By analyzing the shape, size, location, and distribution of bloodstains through bloodstain pattern analysis, it is possible to estimate the types and reconstructions of actions that occurred during the crime [3].

**FIGURE 1:**
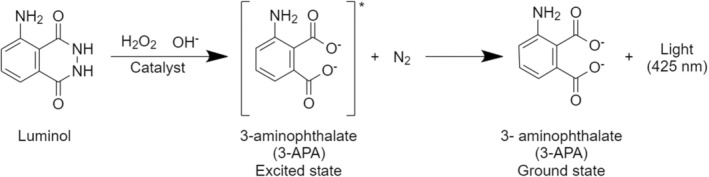
Luminol chemiluminescence reaction scheme.

Bloodstains at crime scenes are often removed or diluted, making them invisible to the naked eye. Detecting these latent bloodstains is crucial for solving a case. Bloodstains on dark clothing or backgrounds are particularly challenging to detect with the naked eye, requiring the application of chemical reagents to enhance their visibility. Luminol reagents are commonly used in such scenarios, as they can reveal erased or diluted bloodstains. Luminol ($C_{8}H_{7}N_{3}O_{2}$, 5‐amino‐2,3‐dihydrophthalazine‐1,4‐dione) is a highly sensitive chemical used for blood detection, capable of identifying blood traces at dilutions up to 1:10,000,000 [4, 5]. When luminol interacts with hemoglobin, the iron within hemoglobin catalyzes the redox reaction, oxidizing luminol. During this process, luminol molecules are converted into a dianion form, which further accelerates the oxidation reaction. As the dianion undergoes oxidation, it forms an unstable compound in a triplet state. Upon returning to its ground state, the luminol derivative emits blue fluorescent light (approximately 425 nm) [6, 7, 8, 9] (Figure 1). These characteristics make luminol an invaluable tool for visualizing latent bloodstains [10].

In forensic research and education, artificial blood substitutes are frequently used instead of human blood for convenience and safety. These substitutes are typically categorized into two types based on their function: synthetic blood and spatter blood [11]. Synthetic blood reacts with luminol but differs from human blood in physical properties such as viscosity, viscoelasticity, and surface tension. Spatter blood, on the other hand, is used for reproducing bloodstain patterns but does not react with luminol. The functional separation of luminol reaction functionality and bloodstain pattern reproduction in current artificial blood substitutes limits the simultaneous analysis of both properties, reducing the reliability of forensic experiments and educational applications.

To overcome these limitations, a new artificial blood (referred to as YFBS) has been developed. This substitute not only responds to luminol but also closely mimics the rheological properties of human blood [11]. Impact spatter patterns, formed by the radial or irregular dispersion of blood due to external forces, are useful for analyzing the hydrodynamic properties of blood, such as viscosity and viscoelasticity. As such, impact spatter patterns are commonly employed to evaluate bloodstain morphology [12]. This study aimed to validate both the luminol reaction functionality and the morphological accuracy of YFBS by comparing its performance to existing artificial blood substitutes.

**FIGURE 2:**
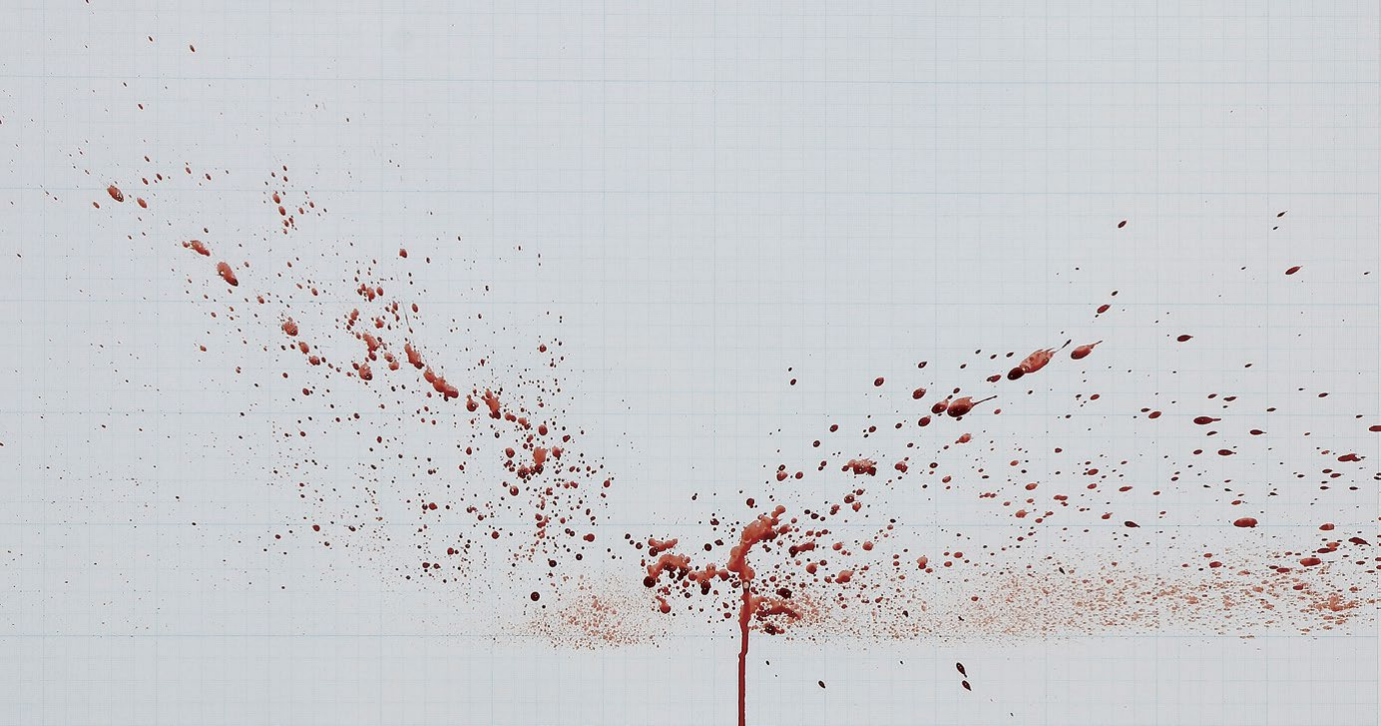
Impact spatter pattern generated using YFBS.

The area of origin refers to the space in three dimensions to which the trajectories of spatter can be utilized to determine the location of the spatter‐producing event [13] (Figure 1). By analyzing the trajectories and angles of individual bloodstains, the area of origin allows for the estimation of the region where blood droplets dispersed due to the external force. Estimating the area of origin is crucial for understanding impact spatter patterns, as this area represents the point where blood was impacted, leading to the formation of the spatter pattern [14, 15]. Methods for estimating the area of origin include the string method, specialized software like Hemospat(FORident Software, Inc. USA), and 3D scanning techniques [15, 16]. The string method, commonly employed in court and public demonstrations, calculates the impact angle of individual bloodstain droplets (Figure 2) and determines the convergence area by connecting lines according to these angles [17].

In this study, we address the limitations of existing artificial blood substitutes, which are typically divided into spatter blood for replicating bloodstain patterns and synthetic blood for studying specific blood properties like luminol reactivity. To overcome these constraints, we examine the newly developed YFBS, which combines rheological properties closely resembling human blood with luminol reactivity. We aim to verify its luminol reaction functionality by comparing its reaction intensity to four types of synthetic blood and further evaluate its morphological accuracy by analyzing the area of origin characteristics for human blood, three types of spatter blood, and YFBS. By integrating both luminol reactivity and bloodstain pattern reproduction capabilities into a single artificial blood substitute, this study seeks to enhance the accuracy, reliability, and versatility of bloodstain pattern analysis in forensic education and research.

## 2 MATERIALS AND METHODS

### 2.1 Comparison of luminol reaction functionality

Four types of synthetic blood were selected for comparison with YFBS in terms of luminol reaction intensity. These synthetic blood types, sourced from Arrowhead Forensics(USA), Evident(USA), Sirchie(USA), and Tritech Forensics(USA), were stored at a room temperature of 20°C. To ensure freshness, the developed YFBS was produced on the day of the experiment and used immediately at 20°C.

Bluestar® forensic(Bluestar, USA), a luminol‐based reagent, was chosen for this study due to its stability, ease of use, and strong luminous intensity [18, 19]. The Bluestar® forensic reagent was prepared by dissolving two types of tablets, one beige and one white, in 125 mL of distilled water. The beige tablet, a chemiluminescent tablet, consists of Sodium Hydroxide(NaOH), citric acid, 5‐amino‐1,2,3,4‐tetrahydrophthalazzine‐1,4‐dione, and polyethylene glycol. In contrast, the white tablet, composed of hydrogen peroxide–urea oxidizer and polyethylene glycol, facilitates the oxidative reaction. The Bluestar® forensic solution was prepared and used on the day of the experiment to ensure freshness and was stored at room temperature (20°C).

Various fabrics, including clothing, are commonly found at crime scenes, with the clothing of both the perpetrator and the victim often collected as evidence. Accordingly, fabrics were utilized as experimental materials in this study. Moreover, as the aim of the experiment was to compare the fluorescence of different artificial bloods with luminol, dark‐colored fabrics were chosen to enhance the visibility of the fluorescence. A black cotton fabric (thread thickness: 20, Enjoy Home company) that readily absorbs blood was selected as the sample for dispensing artificial blood. To minimize noise in the luminescence images, the fabric color was black. The fabric was cut into 1 × 1 cm squares for the experiments. After dispensing YFBS, the four types of synthetic blood, and the Bluestar® forensic reagent onto the fabric, the luminescence reaction was observed.

The luminescence images were captured using the IVIS® Lumina XR imaging system(PerkinElmer, Inc. USA), which can measure bioluminescence and fluorescence using a high‐sensitivity CCD camera to minimize noise. This system visualizes the specific structure or location of fluorescent substances, providing fluorescence imaging. To maintain consistency during experiments, internal standard samples such as calibration beads, which provide appropriate signal intensities across various wavelengths, and luciferase solutions were used to compare and calibrate the intensity and distribution of the fluorescence. For the experiments, 20 μL of each type of artificial blood was uniformly dispensed onto the prepared fabric sample, followed by 100 μL of Bluestar® forensic reagent. The luminous intensity was measured immediately after dispensing. To ensure the reliability of the results and enable quantitative analysis, images were captured 12 times for each type of artificial blood. The images were photographed in bioluminescence mode. The area of the 1 cm^2^ square fabric sample was selected as the region of interest (ROI), and the average radiance (photon/s/cm^2^/sr) from the bioluminescence imaging was used for comparison and analysis.

### 2.2 Comparison of coordinate characteristics of the area of origin of impact spatter pattern

**FIGURE 3:**
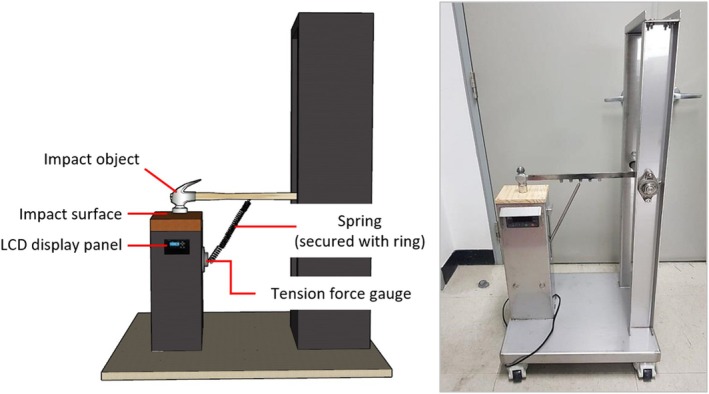
Device for generating impact spatter pattern.

To verify the morphological characteristics of YFBS, human blood and three types of spatter blood (Arrowhead Forensics, Sirchie, and Tritech Forensics) were used for comparison and in the analysis of the coordinate characteristics of the area of origin of impact spatter. The human blood used in the experiment was whole blood drawn from the vein of a 30‐year‐old male, with a hemoglobin(Hb) level of 14.8 g/dL and a packed cell volume(PCV) of approximately 44.4%. For several experiments, blood was collected in EDTA tubes and stored for up to 48 h at room temperature(20°C). All blood samples, including YFBS, were maintained at the same temperature. An impact spatter pattern generating device was designed using a load cell to apply a constant force, ensuring uniform impact spatter for comparison and analysis (Figure 2) [11]. To generate impact spatter, 2 mL of blood at 20°C was dispensed onto the impact point, and a force of 30 kg was applied. The target distance from the impact point was fixed at 30 cm, and the impact spatter pattern was generated on a white wall. This method was used for generating impact spatter patterns from human blood, the three types of spatter blood, and YFBS (Figure 3). The impact angle was measured to calculate the area of origin, and the similarities among the blood types were compared. Based on previous research, at least four individual bloodstains should be selected when estimating the area of origin from spatter patterns [12]. In this study, a total of 12 individual bloodstain droplets (6 droplets each from the left and right) were selected for analysis. The area of origin was calculated using Hemospat software. To ensure the reliability of the data, 10 trials were conducted for each blood type under the same conditions, resulting in 50 total experiments. The average coordinates (*X*, *Y*, and *Z* axes) of the area of origin were calculated and analyzed for each blood type.

## 3 RESULTS

### 3.1 Verification of luminol reaction functionality

The luminous intensity of each blood sample was measured 12 times using the IVIS® Lumina XR imaging system. The mean radiance values (photon/s/cm^2^/steradian; sr) and standard deviations (SD) for the four synthetic blood samples and YFBS are presented in (Table 1). The mean values were as follows: Arrowhead Forensics, 4.850 photon/s/cm^2^/sr (SD = 0.579); Evident, 4.744 photon/s/cm^2^/sr (SD = 0.838); Sirchie, 4.234 photon/s/cm^2^/sr (SD = 0.626); Tritech Forensics, 4.577 photon/s/cm^2^/sr (SD = 0.933); and finally YFBS, 3.226 photon/s/cm^2^/sr (SD = 0.302).

**TABLE 1:** Mean and standard deviation value of average radiance value (photon/s/cm^2^/sr) for four types of synthetic blood and YFBS.

Blood	Average radiance (photon/s/cm^2^/sr) (unit: *e* ^10^)				
	Arrowhead	Evident	Sirchie	Tritech	YFBS
Mean	4.4850	4.744	4.234	4.577	3.226
SD	0.579	0.838	0.626	0.933	0.302

These results demonstrate that YFBS exhibits a lower mean radiance compared to the other synthetic blood types. However, it also shows the smallest standard deviation, indicating a higher degree of consistency and stability in luminescence measurements. This consistency is crucial for ensuring reliable and reproducible results in forensic bloodstain pattern analysis, highlighting YFBS as a potentially valuable tool for such applications.

### 3.2 Results of comparison of coordinate characteristics of area of origin of impact spatter pattern

**FIGURE 4:**
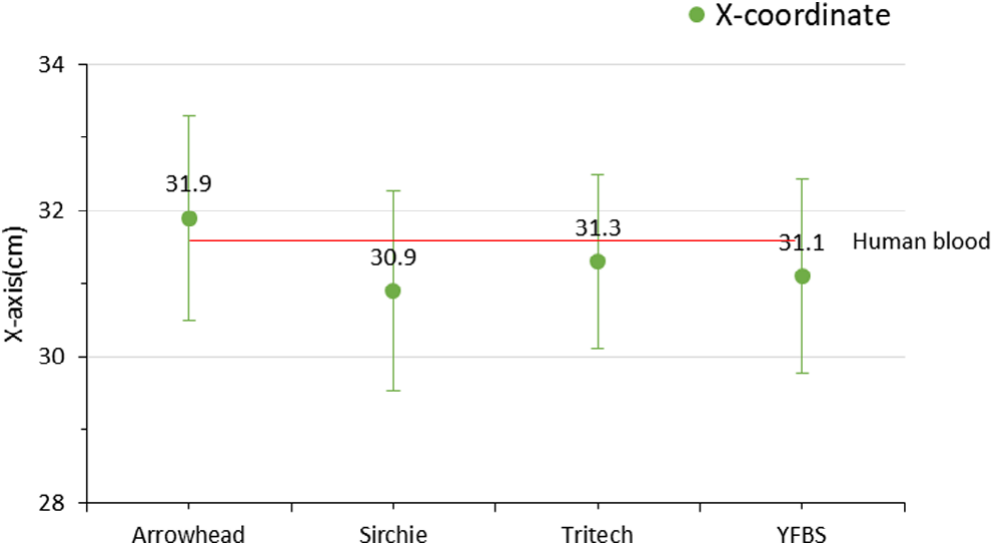
*X*‐axis coordinates of the area of origin for human blood, three types of spatter blood, and YFBS.

**FIGURE 5:**
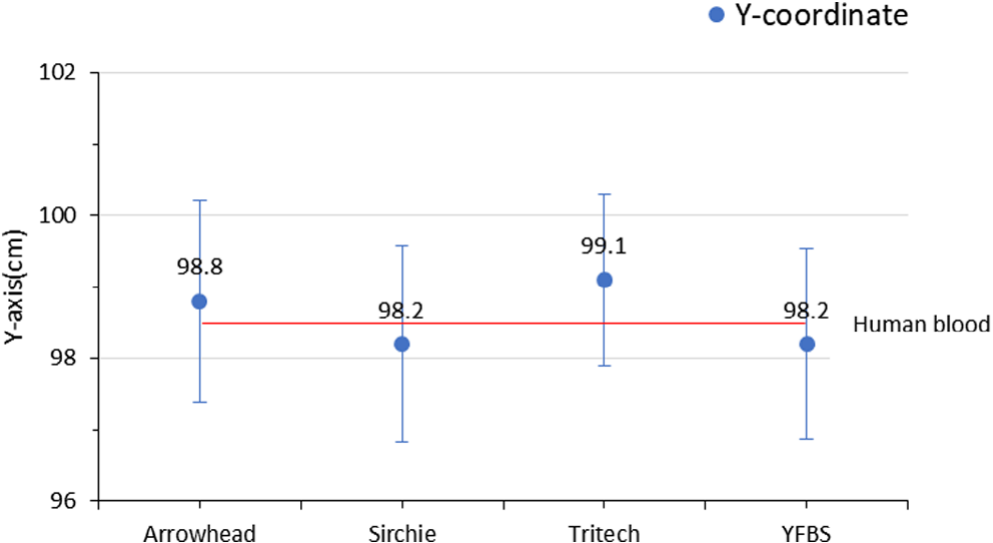
*Y*‐axis coordinates of areas of origin for human blood, three types of spatter blood, and YFBS.

**FIGURE 6:**
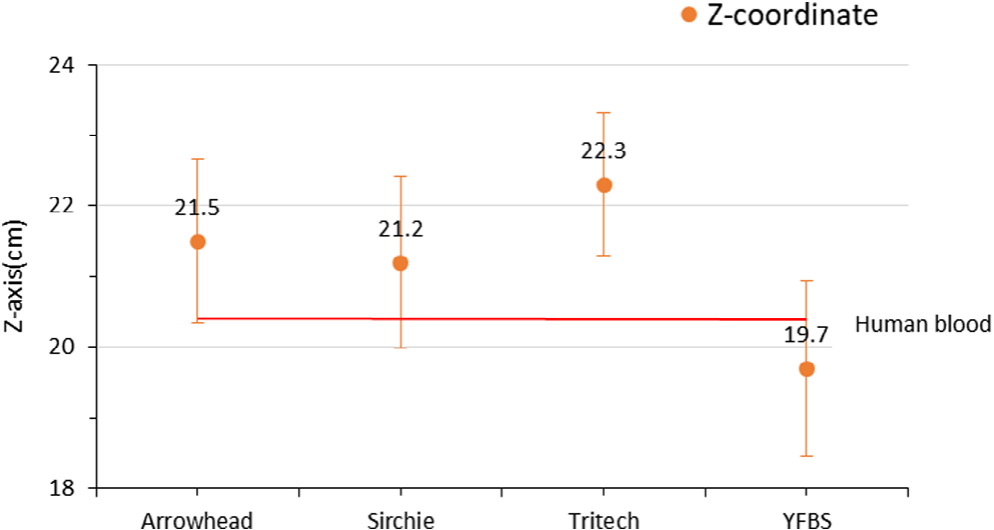
*Z*‐axis coordinates of area of origin for human blood, three types of spatter blood, and YFBS.

To assess the morphological characteristics of bloodstains, impact spatter patterns were generated, and the areas of origin were estimated using the impact angles of individual bloodstains along the *X*, *Y*, and *Z*‐axis coordinates. A total of 50 impact spatter patterns were analyzed, with 10 patterns generated for each blood type. The actual coordinates of the blood spatter origin were set at 30.0 cm (*X*‐axis), 96.7 cm (*Y*‐axis), and 18.6 cm (*Z*‐axis). Following spattering, the estimated coordinates for human blood were 31.6, 98.5, and 20.4 cm for the *X*, *Y*, and *Z*‐axes, respectively. The estimated coordinates of the area of origin for the spatter blood types and YFBS are summarized in (Table 2), with the mean values calculated and compared in Figures 4, 5, 6. Standard deviation values were calculated for human blood, the three commercial spatter blood types (Arrowhead Forensics, Sirchie, and Tritech Forensics), and YFBS to determine coordinate error rates between the actual and estimated areas of origin. The coordinate percent errors for each type along the three axes were as follows:

- *X*‐axis: Human blood (5.3%), Arrowhead Forensics (6.3%), Sirchie (3.0%), Tritech Forensics (4.3%), and YFBS (3.6%).
- *Y*‐axis: Human blood (1.9%), Arrowhead Forensics (2.1%), Sirchie (1.5%), Tritech Forensics (2.4%), and YFBS (1.5%).
- *Z*‐axis: Human blood (9.7%), Arrowhead Forensics (15.5%), Sirchie (13.9%), Tritech Forensics (19.8%), and YFBS (5.9%).

**TABLE 2:** Data on the coordinate characteristics of the impact spatters area of origin for each blood substitute.

No.	Blood type	True value axis (cm)			Estimation origin of area axis (cm)			
		*X*	*Y*	*Z*		*X*	*Y*	*Z*
1	Arrowhead	30	96.7	18.6	Mean	31.9	98.8	21.5
				
Std	0.55	0.41	0.56
2	Sirchie				Mean	30.9	98.2	21.2
				
Std	0.37	0.37	0.62
3	Tritech				Mean	31.3	99.1	22.3
				
Std	0.42	0.20	0.41
4	YFBS				Mean	31.1	98.2	19.7
				
Std	0.55	0.33	0.64

The overall mean percent errors across all axes were calculated as follows: Human blood (5.63%), Arrowhead Forensic (7.96%), Sirchie (6.13%), Tritech Forensics (8.83%), and YFBS (3.6%). These findings reveal minimal differences between spatter blood types and YFBS when compared to human blood. Notably, YFBS exhibited the smallest percent error, particularly in the *Z*‐axis coordinate, highlighting its morphological similarity to human blood and its potential as a reliable substitute for forensic bloodstain pattern analysis.

## 4 CONCLUSIONS AND DISCUSSION

In this study, the Bluestar® forensic reaction intensities of four types of synthetic blood and YFBS were measured and analyzed through luminescence imaging. Additionally, the morphological characteristics of the impact spatter patterns for human blood, three types of spatter blood, and YFBS were evaluated by comparing the coordinate characteristics of their respective areas of origin.

**FIGURE 7:**
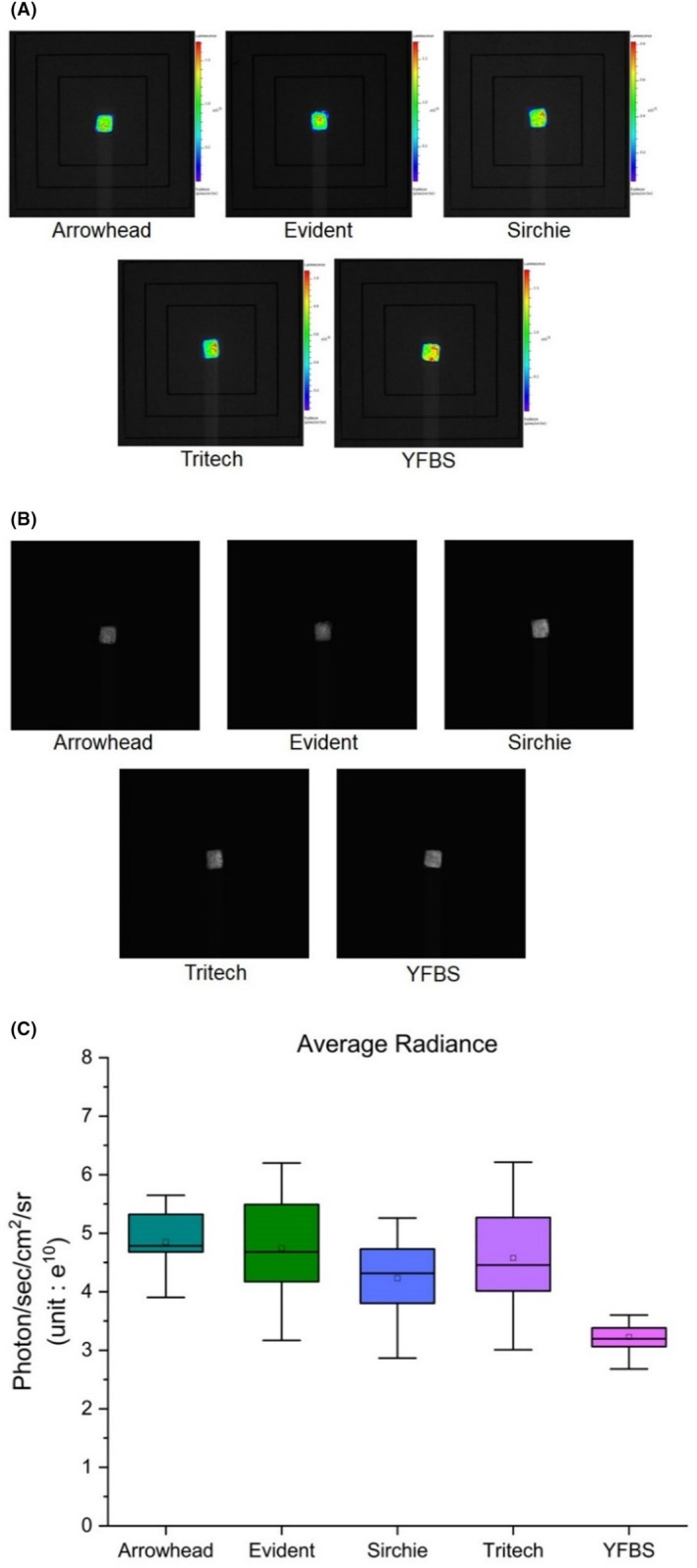
Comparison of average radiance of four types of synthetic blood and YFBS (A) Bioluminescence image by IVIS Lumina XR Imaging System, (B) Luminescent image by IVIS Lumina XR Imaging System, (C) Average radiance(photon/s/cm^2^/sr), and error graph.

Among the synthetic blood types, the sample from Arrowhead Forensics exhibited the highest luminous intensity when reacted with Bluestar® forensic reagent. However, the differences in luminous intensity among the other synthetic bloods and YFBS were not substantial. In practical applications, luminol reactions at crime scenes are typically observed under darkroom conditions. Under these circumstances, luminescence imaging—much like naked‐eye observation—shows limited differentiation in reaction intensities among blood types (Figure 7B). YFBS displayed the smallest standard deviation, indicating its consistent and stable luminescence measurements, which is a crucial advantage over existing synthetic bloods. The use of the IVIS® Lumina XR imaging system facilitated precise measurement, as luminescence images were captured within approximately 2 s after reagent application. Prior research has confirmed that luminol reactions remain measurable up to 3 s post‐dispensing, supporting the reliability of this experimental setup [20, 21].

The coordinate analysis of the area of origin for impact spatter patterns revealed no significant differences between human blood, the three types of spatter blood, and YFBS. This suggests that YFBS possesses the rheological properties required for accurate reproduction of bloodstain patterns while also retaining luminol reactivity. The limited variation in coordinate error rates could be attributed to the short distance between the area of origin and the bloodstain attachment surface, which restricted the bloodstain droplets from exhibiting significant projectile motion. Notably, YFBS showed the smallest coordinate error rate, particularly in the *Z*‐axis, underscoring its accuracy in replicating the morphological characteristics of human blood. It is expected that under conditions where the area of origin is farther from the target surface—allowing for projectile motion influenced by external forces such as gravity—YFBS will demonstrate an even closer resemblance to human blood than existing spatter bloods. These findings collectively suggest that YFBS provides a promising alternative for forensic bloodstain pattern analysis, offering advantages in both luminol reactivity and morphological accuracy.

Further research and experiments will focus on enhancing the luminol reaction functionality of YFBS and expanding its applicability in forensic science. Specifically, efforts will aim to improve the sensitivity and consistency of the luminol reaction, ensuring reliable detection of bloodstains under various challenging conditions, such as trace or highly diluted bloodstains often encountered at crime scenes. In this context, we also plan to investigate the maximum dilution ratio at which luminol reactivity remains detectable, providing critical insights for practical forensic applications. Simultaneously, advanced formulations of YFBS will be developed to achieve both exceptional luminol reaction intensity and highly accurate bloodstain pattern reproducibility. This will involve detailed evaluations of YFBS's rheological properties, including viscosity, flow behavior, and surface tension, ensuring that these characteristics closely mimic those of human blood. The similarity between YFBS and human blood will also be validated using statistical methods. An *F*‐test will first confirm the homogeneity of variance between datasets, followed by a Student's *t*‐test to assess the statistical significance of similarities in measured properties. These analyses will provide a robust framework for evaluating the reliability and applicability of YFBS in forensic science, ensuring it serves as an effective substitute for human blood in both luminol‐based detection and bloodstain pattern analysis. By addressing these critical aspects, this research seeks to establish YFBS as a versatile and reliable artificial blood substitute, capable of enhancing forensic education, research, and real‐world crime scene investigations.

## CONFLICT OF INTEREST STATEMENT

To the best of our knowledge, the named authors have no conflict of interest, financial or otherwise.
